# Management of an Uncorrected Tetralogy of Fallot for Caesarean Section Using Low-Dose Combined Spinal Epidural Anaesthesia Under Advanced Haemodynamic Monitorization

**DOI:** 10.5152/TJAR.2022.21388

**Published:** 2022-08-01

**Authors:** Nuray Camgöz Eryılmaz, Gökçen Emmez, Bedirhan Keskin, Özge Arabacı, Berrin Günaydın

**Affiliations:** 1 Department of Anaesthesiology and Reanimation, Gazi University Faculty of Medicine, Ankara, Turkey

**Keywords:** Caesaren delivery, combined spinal epidural anaesthesia, haemodynamic monitoring, tetralogy of Fallot

## Abstract

Since management of parturients with uncorrected tetralogy of Fallot reported until now lacks advanced cardiac haemodynamic monitoring, we aimed to present anaesthetic management of a parturient with uncorrected tetralogy of Fallot scheduled for caesarean section by addressing the challenges in the management based on the advanced haemodynamic monitoring due to the expected high-risk maternal morbidity and mortality in this particular case. Hereby, we provided haemodynamic stability with little requirement for vasopressor medication by using low-dose combined spinal epidural anaesthesia in a parturient with uncorrected tetralogy of Fallot scheduled to undergo caesarean delivery.

**Main Points**

- Management of parturients with uncorrected tetralogy of Fallot (ToF) via advanced cardiac haemodynamic monitoring provided haemodynamic stability.
- Catheter-based neuraxial techniques, either conventional lumbar epidural or combined spinal epidural anaesthesia, are more favourable than a single-shot spinal block in cardiac pregnant women.
- We used uterotonic drugs in patients with uncorrected ToF who underwent caesarean section.

## Introduction

Natural survival of tetralogy of Fallot (ToF) patients without corrective procedure into the third to fourth decade is extremely rare. However, pregnancy in women with uncorrected ToF was observed at a rate of 1.4/10 000 in West China.^1^ In case of pregnancy of ToF patients, modified World Health Organization (WHO) classification of maternal cardiovascular risk category is III, which shows severe maternal morbidity and increased mortality. As for corrected ToF in pregnancy, modified WHO’s pregnancy risk category is II showing moderate morbidity and low-risk maternal mortality.^2^ A couple of cases with uncorrected ToF for vaginal delivery and low-dose combined spinal epidural (CSE) for caesarean delivery have been reported.^3,4^ We aimed to present management of a high-risk parturient with uncorrected ToF who underwent caesarean section (CS) by addressing the challenges in the anaesthesia management based on the advanced haemodynamic monitoring.

## Case Presentation

**Figure 1.**
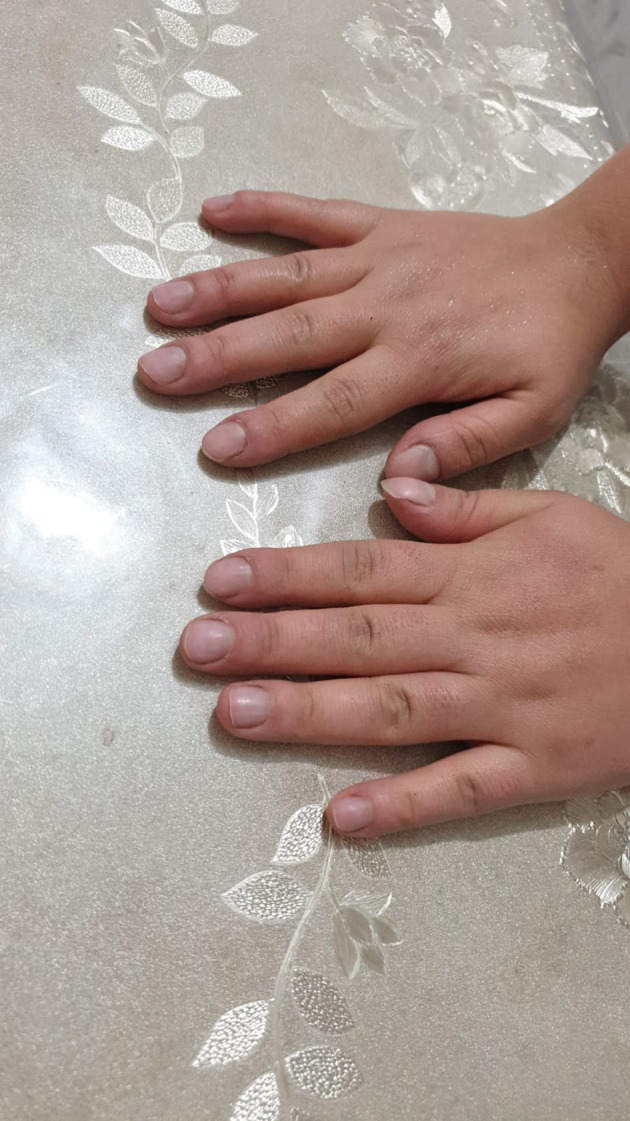
Clubbing of fingers.

A 21-year-old parturient having American Society of Anesthesiologists (ASA) IV physical status (G1, P0, body mass index: 26.4 kg m^−2^) was performed CS at 35 weeks’ gestation after obtaining her written permission. On physical examination, she had clubbing in all fingers (Figure 1), poor exercise tolerance, and pansystolic murmur on auscultation. According to echocardiography (ECG) at 25 weeks’ gestation, aortic valve dextroposition, ventricular septal defect (VSD) (1.4 cm) in the basal of interventricular septum, dilated and hypertrophic right ventricle (RV), right ventricular outflow tract (RVOT) max. 82/mean, 44 mm Hg systolic gradient, and ejection fraction of 65% were determined. On 28 weeks’ gestation, ECG was normal sinus rhythm, but tachycardia attacks were recorded by holter during the day. At 33 weeks, she was admitted to the cardiology ward with New York Heart Association (NYHA) class III and modified WHO pregnancy risk category III to monitor closely until delivery. Preoperative complete blood count (haemoglobin 14.5 g dL^−1^, hematocrit (Htc) 44%, and platelet count 209 000 mm^-^^3^) and biochemistry results including creatine kinase MB (CK-MB) were unremarkable, but ferritin and transferrin saturation was 13.9 ng mL^−1^ and 10%, respectively.

After intravenous (iv) metochlopramid (10 mg), initial standard monitorization including heart rate, ECG, peripheral oxygen saturation (SpO_2_), and non-invasive blood pressure (BP) was established and followed by continuous invasive BP monitoring via radial artery cannulation. Then, advanced haemodynamic parameters were invasively and continuously monitored to follow the patient until the post-operative period (Mostcare up, Pressure Recording Analytical Method, Vygon, UK).

**Figure 2.**
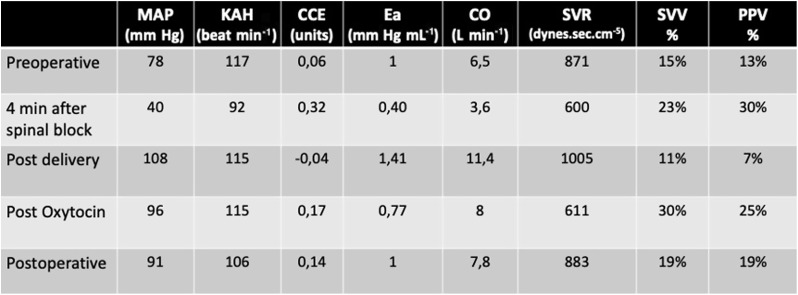
Advanced hemodynamic parameters. MAP, mean arterial pressure; HR, heart rate; CCE, cardiac cycle efficiency; Ea, arterial elastance; CO, cardiac output; SVR, systemic vascular resistance; SVV, stroke volume variation; PPV, pulse pressure variation.

The CSE was performed between L3 and L4 intervertebral space in the sitting position using needle through needle technique. Intrathecal hyperbaric bupivacaine of 5 mg (Bupivon, heavy 0.5%, 4 mL ampule, ONFarma, Ankara), 20 μg of fentanyl (Fentaver 0.5 mg 10 mL^−1^, Haver, İstanbul), and 100 μg of morphine (Morphine HCl, 10 mg mL^−1^, Galen İlaç, İstanbul) were administered and then epidural catheter was placed. Since SpO_2_ was 85% while breathing room air, 2-5 L min^−1^ of supplementary nasal O_2_ was given. Four minutes after neuraxial block, when BP and systemic vascular resistance (SVR) significantly decreased, 5 μg of iv noradrenaline (diluted solution which was ready to use) was administered. When sensory block reached to thoracal 4 dermatom, onset of surgery was allowed. Three minutes after skin incision, a male baby (2310 g, 44 cm) was born. Newborn’s Apgar scores were 7 and 9 at 1 and 5 minutes, respectively. After delivery, iv furosemide (20 mg) was given. Meanwhile, oxytocin (10 IU 500 mL^−1^ saline) infusion (2.4 IU 15 min^−1^) was started initially followed by 2.4 IU h^−1^. Afterwards, SVR decreased but returned to normal values without any further medication (Figure 2). The operation lasted 45 minutes. Paracetamol (1 g) iv and neuraxial morphine provided satisfactory analgesia until discharge. Epidural catheter was not used during and after the operation. It was removed at post-operative 24 hours. No complication associated to anaesthesia was encountered.

## Discussion

We have presented CSE anaesthesia/analgesia management of a parturient with uncorrected ToF who underwent CS under invasive advanced haemodynamic close monitoring, which has been the first case report in this respect.

Choice of anaesthesia for CS of most uncomplicated healthy pregnant patients is neuraxial anaesthesia (spinal, epidural, or CSE) rather than general anaesthesia because of the opportunity to avoid general anaesthesia-related risks, mainly due to difficult airway. Among neuraxial techniques, spinal block has been the most common one despite the onset of block being associated with hypotension resulting from decreased venous tone and SVR which might be very much problematic in cardiac pregnant women. Additionally, spinal block associated with decreased SVR causes rapid cardiopulmonary decompensation than that of epidural or CSE.^5^ Therefore, in parturients with congenital cardiac disease CSE, using opiods may be optimal.^2^ As a result, catheter-based neuraxial techniques, either conventional lumbar epidural or CSE, are more favourable than a single-shot spinal block to provide segmental anaesthesia/analgesia more reliably due to the gradual haemodynamic changes by dose titration. Currently, we performed CSE using fentanyl and morphine and managed it successfully by the support of close monitoring of advanced haemodynamic cardiac parameters.

In the management of spinal anaesthesia-induced hypotension, phenylephrine is regarded as first-line vasopressor in patients undergoing CS.^6^ However, similar effectiveness with lower number of physician interventions and less reactive hypertension and bradycardia have been shown when noradrenaline vs phenylephrine was compared. Hence, noradrenaline has been a suitable alternative to phenylephrine.^7^ In corrected ToF cases who underwent CS using CSE or conventional epidural, phenylephrine was used to treat hypotension.^8^ In contrast to this practice, we preferred 5 μg of iv noradrenaline based on the estimated ED90 dose (5.49-5.80 µg) as an alternative vasopressor.^7, 9^

It is controversial to use uterotonic drugs in patients with uncorrected ToF who underwent CS, either uterotonic use was avoided^4^ or methylergonovine was used,^9^ because perioperative myocardial ischemia and post-operative death due to Eisenmenger’s syndrome were reported when methylergonovine was used.^9^ Since oxytocin was used for labor induction uneventfully in the pregnancy management of an uncorrected ToF case,^3^ we similarly used iv oxytocin infusion after delivery with careful titration due to its potential risk to decrease SVR. Hereby, SVR restoration after oxytocin was achieved by noradrenaline.

## Conclusion

Management of parturients with uncorrected ToF reported until now lacks advanced cardiac haemodynamic monitoring. Successful haemodynamic stability in a parturient with uncorrected ToF was provided with a little vasopressor requirement by using low-dose CSE for CS.
